# Admission Decision-Making in Hospital Emergency Departments: The Role of the Accompanying Person

**DOI:** 10.1177/2333393620930024

**Published:** 2020-06-18

**Authors:** Susanna Rance, Debra Westlake, Heather Brant, Ingrid Holme, Ruth Endacott, Jonathan Pinkney, Richard Byng

**Affiliations:** 1 University of East London, London, United Kingdom; 2 University of Plymouth, Plymouth, United Kingdom; 3 University Hospitals Bristol NHS Foundation Trust, Bristol, United Kingdom; 4 University College Dublin, Dublin, Ireland; 5 Monash University, Frankston, Victoria, Australia

**Keywords:** carers, caregivers, emergency department, emergency room, decision-making, ethnography, qualitative research, United Kingdom

## Abstract

In resource-stretched emergency departments, people accompanying patients play key roles in patients’ care. This article presents analysis of the ways health professionals and accompanying persons talked about admission decisions and caring roles. The authors used an ethnographic case study design involving participant observation and semi-structured interviews with 13 patients, 17 accompanying persons and 26 health care professionals in four National Health Service hospitals in south-west England. Focused analysis of interactional data revealed that professionals’ standardization of the patient–carer relationship contrasted with accompanying persons’ varied connections with patients. Accompanying persons could directly or obliquely express willingness, ambivalence and resistance to supporting patients’ care. The drive to avoid admissions can lead health professionals to deploy conversational skills to enlist accompanying persons for discharge care without exploring the meanings of their particular relationship with the patients. Taking a relationship-centered approach could improve the attention to accompanying persons as co-producers of health care and participants in decision-making.

## Introduction

Hospital emergency departments (EDs)—also known as accident and emergency departments, emergency rooms or casualty departments—are the main entry point to acute care in many health systems. In the National Health Service (NHS) in England, they offer unscheduled access for patients who self-present or are referred from primary or secondary care. People accompanying patients in ED visits, such as partners, relatives, and friends, can play key roles in giving health professionals background information and helping to make decisions about patient care. They may also be drawn upon as a “hidden workforce” performing tasks for patients that may otherwise be carried out by nurses (Fry et al., 2015), particularly in contexts of limited staffing (Bridges et al., 2010; Gordon et al., 2010). Relatives may also be involved in ensuring patient safety in hospital (Merner et al., 2019).

Relationships between patients and carers (or caregivers, a term more commonly used in the U.S.A.) are influenced by multiple factors, including the patient’s medical and health history and the dynamic nature of illness and family situations, which may mean that caring roles and responsibilities change over time (Swinkels et al., 2018). Despite this variation, in studies of hospital settings including EDs, analyses of carers’, relatives’ and patients’ views and experiences are often combined. The use of dyadic categories such as “patients and carers” or “patients and relatives” can convey an assumption that all parties involved are equally focused on the fulfillment of the patients’ needs (Doos et al., 2014; Grimmer et al., 2006; Rainey et al., 2013). Referring collectively to such parties as though they were united in their interests can create a sometimes unwarranted impression of established relationships and caring arrangements. Recent evidence suggests that, for adult patients in particular, caring relationships cannot be defined *a priori.* Rather, these are negotiated case by case in processes that may involve carers, the patient and health professionals (Aasbø et al., 2016; Allen, 2000; Finch & Mason, 2003). The United Kingdom (U.K.) legislation and policy directives refer to carers’ involvement in decision-making about patients’ care (Care Act, 2014). However, this guidance is inconsistently followed (Wingham et al., 2016). Relatives and other caregivers value being included in decision-making (Fry et al., 2015; Karnieli-Miller et al., 2012; Laidsaar-Powell et al., 2013; Nikki et al., 2012). Professionals sometimes experience their interactions with patients’ relatives as challenging, for example, when relatives intervene on behalf of patients, or when they disagree with medical advice (Fry et al., 2015; Laidsaar-Powell et al., 2013; Pinkney et al., 2016).

EDs in England are increasingly resource-strained and affected by crowding (Department of Health, 2010; Higginson, 2012). During a 2013 to 2014 mixed-methods organizational case study of ED decision-making in four NHS hospitals across south-west England, the authors of this article found that ED practitioners were temporally constrained by the NHS 4-hour target for decision-making about patients’ admission or discharge. They were also administratively driven by contractual penalties that are charged to hospitals when this target is breached (Department of Health, 2010; Pinkney et al., 2016; Weber et al., 2011). Among findings from the ethnographic component of our wider organizational study, we observed instances where caring relationships were being negotiated in the reduced time-frame characteristic of the ED setting (Pinkney et al., 2016).

Our study sites used different models of emergency care, but all deployed multiple top-down and ground-up initiatives to avoid unnecessary acute admissions. Some relatives and carers expressed views about patients’ admission or discharge that differed from those of health professionals. Patients and relatives sometimes disagreed among themselves about whether or not the patient should be discharged home. Their considerations included not only how the patient could be affected by staying longer in hospital, but also implications for the different people who might be involved in practical arrangements for home care (Pinkney et al., 2016).

Regarding the question of defining carers, it is now recognized that the use of this term has been over-standardized. Traditionally, sociological analyses of carers’ roles have conceptualized these as instrumental to the maintenance of institutional systems, often influenced by normative expectations about a duty of family support (Twigg & Atkin, 1994). More recently, self-identification with carer roles and labels has been found to be “nuanced, shifting and variable” (Hughes et al., 2013). Not all people performing caring tasks consider themselves to be carers (Orr et al., 2013). Some people providing support in a non-professional capacity (Beesley, 2006) represent their helping activities as an intrinsic part of family relationships (Molyneaux et al., 2011). Not all those named by others as carers wish to act in this capacity (NHS England, 2014). A growing body of work examines how people come to name themselves—or resist being labeled—as carers in particular relationships or contexts (Aasbø et al., 2016, 2017; Chattoo & Ahmad, 2008; Molyneaux et al., 2011; Schumm et al., 2010; Twigg & Atkin, 2009).

## Focused Analysis of Cases Involving Accompanying Persons

The findings of our wider study revealed that people accompanying patients in ED visits often wished to be treated as partners in decision-making. However, they were rarely acknowledged in this capacity, especially when the staff considered that the patient’s voice should come first. Those accompanying patients lacked channels to formalize and support requests for their own concerns either to avoid, or conversely argue for, an admission. For example, if they favored a hospital stay for an elderly patient, they could come into conflict with the patient’s wishes and clinical advice (Pinkney et al., 2016).

For the purposes of our focused analysis on this topic, we wished to avoid the automatic use of “carer” labels, as well as the value-laden connotations of other terms such as “companions” (Laidsaar-Powell et al., 2013; Wolff & Roter, 2008). We therefore adopted the neutral concept of “accompanying persons” (APs) (Ekwall et al., 2009), making no *a priori* judgment on whether or not people presenting with patients in ED did so as carers. We carried out fresh, in-depth analysis of our sub-set of cases from the ethnographic study that involved APs. This may be understood as a mode of secondary analysis of self-collected qualitative data (Heaton, 2008), performed by the same team of primary researchers on cases we had reported on in lesser detail in our first publication from the study (Pinkney et al., 2016). The aim of this analysis was to look more deeply into this group of cases involving APs, to answer two new research questions:

**Research Question 1.** How did APs present themselves and their relationships with patients to health professionals and researchers during ED visits?
**Research Question 2.** How did APs and ED practitioners negotiate issues relating to admission decisions and caring roles?

## Research Design and Methods

This focused analysis was carried out as part of a wider project that used a multiple case study design for a mixed-methods analysis of decision-making about admissions in four acute hospitals in south-west England (Pinkney et al., 2016). The study investigated how clinician expertise and models of care in the four hospitals contributed to decision-making regarding acute admissions. This involved investigating influences operating on the decision process and how the process was experienced by patients and health professionals.

The wider study generated ethnographic data from the four hospital EDs and associated observation and decision-making units. The sites had been selected because of the structural contrasts in the pathways for emergency medical admissions which they were using at the start of the study (Pinkney et al., 2016; Swancutt et al., 2017).

The study recruited medical (non-surgical) participants for whom the clinical decision- makers were not yet certain if admission or discharge was the best option. The participants were aged 18 or older. Patients and relatives were followed through their ED journey. The patients were not clear candidates for any predefined care pathway, and they generally required some clinical observation, investigations, and discussion before an admission or discharge decision could be made (Swancutt et al., 2017). Frequently social issues, such as the presence or absence of support at home or in the community, had to be considered in decision-making. Four researchers [Rance, Brant, Holme, and Westlake] conducted participant observation and semi-structured interviews at all sites between September 2013 and July 2014 during the day, night and weekend shifts, over periods of 6 to 8 weeks in each site. We used purposive sampling to seek maximum variation in the levels and roles of the staff observed and interviewed, and in patients’ characteristics. In the latter part of data collection, we did theoretical sampling of patients to seek a balanced sample by gender and an adequate range of ages and presenting conditions.

Although our ethnography was not designed as a conversation analysis study, our methods were sensitive to language and context, and we were influenced by studies examining the social organization of talk (Atkinson, 1985) and the negotiation of institutional arrangements in spoken interactions (Heritage, 1997). From such studies, we noted that taking a dyadic or triadic approach to observing and interviewing (Adams & Gardiner, 2005; Laidsaar-Powell et al., 2013; Robson et al., 2013) could highlight how participants shifted in their groupings and patterns of convergence or divergence in naturally occurring interactions (“coalition dynamics”) (Biggs et al., 1995; Roscow, 1981). In our focused analysis, we looked closely at APs’ self-positioning, their negotiations with professionals, and the influence of their interventions on decisions taken to admit or discharge patients.

### Participant Observation and Interviews

In the wider study through which our data were generated (Pinkney et al., 2016), written informed consent was given by all parties present in the settings where we made field notes or audio recordings, and for all interviews. We recruited a total of 282 health professionals, 65 patients and 30 “relatives and carers” (as we labeled them at that time). Many of the encounters we observed could be qualified as “naturally occurring,” even though—as is recognized in all forms of qualitative research—the researchers’ presence was inevitably influential.

The ethnographic data set comprised detailed field notes (Emerson et al., 1995) of observations (*n*=107), transcripts of audio-recorded informal conversations and decision-making encounters (*n*=242), and semi-structured interviews with patients, APs, and health professionals (*n*=96). Recording was done in full view of the consented participants, and the researchers alerted anyone entering the room that recording was happening. Audio recordings were transcribed verbatim.

## Analysis

As part of the wider study on ED decision-making, audio transcripts and field notes from the ethnographic data set were independently coded by three researchers [Rance, Brant and Holme] using NVivo10 (QSR International, Warrington, UK). We then merged our coding, agreed on a shared framework, and discussed our analysis to produce findings for the study report (Pinkney et al., 2016).

On the basis of subsequent discussions, together with a fourth researcher [Westlake], we carried out the focused analysis presented in this article to answer specific research questions on APs and their involvement in decision-making encounters. We here report on this new set of findings based on our re-coding of transcripts of interviews and observed encounters involving 13 patients, 17 APs and 26 health professionals (see Table 1 for features of the 13 cases). These 13 patient cases were selected with two inclusion criteria:

1. Presence of one or more APs
2. Availability of ethnographic data from observed interactions as well as recorded interviews (Karnieli-Miller et al., 2012; Kendall et al., 2009).

**Table 1.** Patient Case Studies (*n* = 13) Involving Accompanying Persons (APs): Data From the Interactions Observed in EDs at Four NHS Hospital Sites in South-West England, 2013 to 2014.

Case	Patient Profile^a^	Patient’s Presenting Condition	Admitted or Discharged	AP’s Pseudonym; Relationship With Patient	Health Professionals Observed
1	Male, 83, living with wife	Collapse	Admitted	Agnes-Wife	Foundation Year 1 doctor^b^
2	Male, 73, living in care home	Confusion/funny turn	Admitted	Beatrice-Wife Carol-Daughter	2 Junior doctors^c^, registered nurse from care for the elderly team, and consultant
3	Female, 70, living alone	Dizzy spell/collapse	Discharged	Doris-Daughter 1 Elizabeth-Daughter 2 Son 1, Son 2 (not cited)	Junior doctor, consultant from care for the elderly team, and a consultant in charge of the ED
4	Female, 24, living with boyfriend	Funny turn	Discharged	Frances-Mother	Junior doctor and consultant
5	Female, 41, living alone	Neck pain	Admitted	Graham-Boyfriend	Registered nurse
6	Male, 75, living with wife	Severe headache	Admitted	Harriet-Wife	Junior doctor
7	Female, 82, living in retirement complex	Fall	Admitted	Ian-Son	ED consultant
8	Male, 83, living with wife	Chest pain	Admitted	Judith-Wife	Junior doctor
9	Female, 86, living with partner	Fall/dementia	Admitted	Kenneth-Partner	2 Registered nurses, junior doctor, 2 occupational therapists, consultant
10	Male, 65, living with wife	Query stroke	Discharged	Lucy-Wife	ED consultant and registered nurse
11	Female, 65, living with husband	Fainting/fall	Discharged	Michael-Husband	Junior doctor
12	Male, 86, living with wife	Chest pain	Admitted	Nancy-Wife	Junior doctor and registered nurse
13	Female, 85, living next door to friend	Breathlessness/leg swelling	Admitted	Olivia-Friend/neighbor	Junior doctor

*Note.* EDs = emergency departments.

a All patients in this group were of White ethnicity. South-West England has a higher proportion of White population than many other U.K. regions (Office for National Statistics, 2011). Seven of the 65 patients in our study were self-defined as being of Black and Minority Ethnicity (BME), but their cases did not meet the inclusion criteria for this analysis of AP presence and data from recorded observations as well as interviews.

b A Foundation Year 1 (FY1) doctor is one who has graduated in the past year.

c Since the introduction of the NHS Modernizing Medical Careers (MMC) program in 2005, a junior doctor is one who is still in training 3 to 8 years post-graduation and has not yet reached consultant level. The broad designation of a junior doctor includes “middle-grade” levels and Senior House Officers.

Our fresh coding of these 13 selected case studies produced new analytic categories concerning the place of APs in the ED environment and their disposition toward acting as carers. These led us to identify instances where APs discussed or negotiated tipping points and transitions in their caring status and relationships.

## Ethics

The NHS hospital study sites and identities of patients, APs, and health professionals have been anonymized. NHS ethics and governance approval was granted (Integrated Research Application System reference number 98931 and Research Ethics Committee reference number 12/SW/0173). In our wider study, ethics approval was granted to carry out analyses relating to a broad range of issues regarding admission decisions, including the type of questions investigated in this paper.

## Results

In the sections that follow, we describe features of patient cases involving APs. We then explore new analytic categories concerning the place of APs in the ED environment, including transitions in caring relationships. We examine variations and shifts in the APs’ expressions of willingness, ambivalence, and resistance to being addressed as carers or to taking on additional responsibilities in patients’ forward care. Finally, we consider interactions we observed where APs were discursively “talked into being” as carers.

In labeling the transcript excerpts cited, we differentiate between APs’ declarations made in the presence of health professionals and those made apart to researchers who thus had access to some rich “back-stage” contextualization for the cases (Goffman, 1959; Scott, 1990).

### Patient Cases Involving APs

Table 1 shows features of 13 patient cases. We gave pseudonyms to APs only, to highlight our analytic focus on them rather than on the patients or health professionals. Data on the patients’ mode of arrival to the ED were not collected. Patients and APs were observed in different clinical settings, not detailed in the table.

Of the 13 ED visits involving APs, nine resulted in hospital admissions, and four patients were discharged. We do not claim that these patients’ profiles or outcomes were representative of wider patterns. Rather, our analysis can serve to contextualize APs’ presence, illustrate variability across cases, and examine the workings of the APs’ involvement in decision-making interactions and discussions about caring.

### The Place of APs in the ED Environment

The ED was a liminal space between the context where a patient’s health crisis had occurred and the acute ward, community or home destination following diagnosis. It was spatially bounded as a stopping-point for triage, treatment, and observation. APs were commonly taken by ED practitioners to be a resource that could potentially support discharge strategies, especially for the avoidance of what they labeled “social admissions” (Pinkney et al., 2016) (often responding to a shortfall in home care or support services in the community).

All parties in the interactions we analyzed demonstrated awareness of ED pressures exacerbated by bed shortages and a build-up of cases competing for priority. Health professionals rationed care, and patients and APs were observed to self-ration their demands. APs sought to retain a place in the ED system through strategies such as demonstrating compliance with appropriate behavior, aware that the case of the patient they were accompanying was just one among many:

I had to ask the young nurse there, excuse me what is happening? And she said there is an awful lot of sick people here, so . . . I don’t expect to jump the queue, I don’t . . . [Interview with Harriet (patient’s wife) and the patient. Case 6.]

In ED environments offering little privacy, with small spaces divided by curtained-off cubicles, conversations about patients’ conditions and people’s personal lives were publicly audible. Health professionals called on APs to give information on a patient’s condition, offer practical assistance while awaiting tests, decisions or discharge, provide transport, and back up home care arrangements. Some APs were acknowledged to be good history givers, but as a norm backed by policy, health professionals insisted on hearing directly from patients unless they had significant cognitive impairment. When an AP intervened, the health professional generally kept their attention focused on the patient and continued to speak directly to them. In such exchanges, an AP would not always receive a response from the professional. However, in cases where patients were deemed less able to provide information, some APs told researchers how, exceptionally, their input had been acknowledged:

Carol, patient’s daughter, to researcher: I think what’s been good is that the doctors have actually listened to what we’ve said. [Interview with Carol, Beatrice (patient’s wife) and the patient. Case 2.]

Some APs acknowledged strong ties with patients, but rather than defining themselves as carers they emphasized a particular quality or context of the relational bond:

Ian, patient’s son: We have an even closer bond than a mother and son, because I had renal failure, and mum gave me a kidney, so we’ve got, you know, an extra bond. [Interview with Ian and the patient. Case 7.]

Blood ties could be emulated in non-family relationships, as in the case of Olivia (Case 13), the AP who—without being a blood relative—enacted the most decisively engaged caring role across our cases. She had known the 86-year-old patient for “40-odd years” and had lived next door to her for 26 years. She proactively managed her friend and neighbor’s care, often responded to health professionals and a researcher on her behalf, and identified with her to the point of representing dual candidacy for ED attendance:

Researcher (to patient): So what were you expecting to happen?

Patient: I, well, I really didn’t know.

Olivia (patient’s friend and neighbor, to
researcher): Well I was expecting to come in thinking that they knew that we were coming. . . (. . .) I didn’t visualize coming in here. . . And having all these tests. . .

Researcher (to Olivia): So you know each other quite well then?

Olivia: Very well, yeah . . .

Patient: [laughter] Yeah, we’ve been through a lot. [Like] mother and daughter.

### Relationships in Transition

The encounters we observed took place during the periods in which patients and APs occupied a space in the ED, engaged with health professionals, and sought to influence decision-making. Within the diversity of bonds between the patients and APs, in some cases the ED visit signified a “tipping point” into a new or unforeseen type of caring relationship. Some ED visits were triggered by the first sign of a condition that had not been detected previously. The shock of an initial diagnosis or start to investigations could be compounded by the AP’s sense that the person on whom they had relied was now vulnerable and possibly undergoing a transition in their longer-term health status.

Some APs had not self-presented as carers, but the implications of needing to adjust to such a role started to emerge even as they spoke:

Patient: I run my own small consultancy business. . . . If this is to be a recurring feature I’ll have to wind that business up, I can’t do it.

Lucy, patient’s wife: No. So, in some ways there’s lots of stuff going on around this. . . . you’re in a situation . . . especially when we’re coming into [name of the town] and I’m saying “Um, where do I go here? I don’t know where . . .” . . . Driving his car. An automatic and I don’t drive an automatic.

[Interview with Lucy and the patient. Case 10.]

In other cases, APs described deterioration in the patient’s condition as gradual, but they reflected with researchers on the abrupt transition from a relatively manageable health status to a situation of increased dependency:

Ian, patient’s son: . . . it’s harder and harder for her to manage at home. . . (. . .) I think this fall has. . . tipped it over, you know . . . (. . .) we can’t be there all the time . . . we need to get in more help, do we need Mum to have some respite care, what happens if we’re away . . . [Interview with the patient and Ian. Case 7.]

APs’ Shifting Disposition Toward Acting as Carers. Tangible resource constraints, and ED practitioners’ institutionally mandated collaboration with admission avoidance, added weight to situations in which the APs showed an awareness of normative expectations that they would perform in a caring capacity. In response to the manifest or internalized pressure of such social and institutional norms, we observed how APs could shift between expressing willingness to collaborate with health professionals in supporting the different dimensions of patients’ care; ambivalence when being “cast” by ED practitioners as a carer did not tally with their own sense of a relationship with the patient; and resistance to taking on new caring responsibilities, for example, if an explicit commitment in this direction should be required to enable discharge.

#### Willingness

The ED practitioners’ admission or discharge decisions were sometimes influenced by APs’ proactive moves. This was illustrated in Case 8 where Judith (the patient’s wife) bypassed the couple’s GP (General Practitioner or family doctor) to come straight to the ED, brought the patient in equipped for an overnight stay, and argued successfully for his admission. The presence of a supportive member of the patient’s social network could facilitate a discharge decision, as in Case 5 where Graham (the patient’s new boyfriend) expressed willingness to take on a home care role if this should be needed: “I’m not going back to work until she’s better, I’m not.”

In Case 10, Lucy (a former nurse) identified her husband’s symptoms as typical of a stroke, informed health professionals of her observations, and concurred (as the patient also did) with their decision that he could be safely discharged with an outpatient clinic appointment booked for that afternoon.

#### Ambivalence

As potential or prospective caring activities were discussed, some APs expressed ambivalence about their readiness to adopt a new or augmented role. APs—some new to ED scenarios and others who had experience negotiating the system—spoke more readily to researchers “backstage” than to health professionals about their personal situations and limits to their willingness or capacity for acting as carers. A researcher’s presence was found to act as a catalyst for reflexive comments by all groups of participants (Cant & Sharma, 1998). Some APs used research interactions as an opportunity to talk through problems or reflect on their relationships with patients, possibly finding it more comfortable to talk to the researcher who did not have a stake in decision-making:

Ian, patient’s son, to the researcher: It’s very, very difficult for me to get her to agree to more help. . . . it’s quite a battle, because the roles are reversed now –

Researcher: Yeah.

Ian: I’m telling her what to do – . . . She don’t like it.

Patient: Oh no, I don’t like it at all. [laughter]

(. . .)

Son: When we get older, the roles reverse.

[Interview with patient and Ian. Case 7.]

The situation of being observed could also prompt ED practitioners to demonstrate how they implemented the policy of involving patients and relatives in decision-making:

Consultant: (to the patient) What do you think about that? Would you be happy with that?

Patient: You’re the doctor, you tell me what to do [laughing].

Consultant: No! I’ve got a lady here watching what decisions we make [referring to the researcher]. (To Daughters 1 and 2): What do you think? Does that sound okay? [laughing]

[Recorded observation of the interaction between the consultant for care of the elderly, patient, Doris (Daughter 1) and Elizabeth (Daughter 2). Case 3.]

In this case, the “best practice” behavior mindfully enacted by the consultant consisted of formally involving not only the patient but also her daughters in shared decision-making. However, the consultant’s admission avoidance endeavor did not extend to the more subtle role of exploring differences between the preferences of Elizabeth—who lived near her mother and regularly supported her home care—and Doris, who lived further away and was less affected from day to day by the consequences of her mother’s deteriorating condition.

#### Resistance

We analyzed a set of observed interactions where tension or conflict became apparent in APs’ negotiation of their roles. In such situations, while referring to their own needs, some APs expressed emotions of shame or guilt linked to normative self-/expectations about caring. There were cases where APs—despite giving signs of resistance—were “talked into” accepting caring responsibilities by ED practitioners’ deployment of persuasive conversational skills.

In some cases, APs referred in positive terms to a pre-existing caring relationship with the patient. In others, APs expressed difficulty in asserting their limits to caring, especially when talking with practitioners. Some APs who were patients’ relatives, and particularly partners or spouses, expressed discomfort that seemed to be associated with internalized or externally-applied pressure of normative expectations about caring roles (Swinkels et al., 2018). In such situations, APs could use indirect cues to imply ambivalence or resistance.

#### Shame, guilt, irony, and joking

Given their apparent internalization of social and institutional norms about the duties of informally-provided care, APs in our study rarely expressed overt resistance to taking on greater caring responsibilities. In the course of conversations that were weighted toward the interests of patients and ED system arrangements, they interjected clues about their personal constraints and limits, “leaking” allusions to lifeworld situations (Heritage & Maynard, 2006; Mishler, 1984):

Beatrice, patient’s wife: I think that we have so much going on, with [daughter’s] mother-in-law and my mother . . .

[Interview with Beatrice, Carol (patient’s daughter) and patient. Case 2.]

Kenneth, patient’s partner: I can’t do very much, I’ve not long been out of hospital myself.

[Interview with Kenneth and patient. Case 9.]

Normative expectations could make it difficult for APs to declare or hint at their limits to caring when the patient they were accompanying was a family member. Some APs who had argued for a relative’s admission expressed emotions of shame (blame directed toward the self) or guilt (about a statement or behavior that could be negatively judged) (Tangney et al., 2007), which they immediately refuted in reflexive self-defense:

Carol, patient’s daughter: . . . it makes me feel like that we’re being awful, because we’re not.

[Interview with Carol, the patient, and patient’s wife. Case 2.]

Ian, patient’s son: Not that I want her to be in hospital, it sounds terrible, but I think she’s, at the moment, it’s safer in hospital. . .

[Interview with Ian. Case 7.]

When the patient was a partner or spouse, voicing limits could be still more challenging for APs because of the augmented expectation of a social obligation to care. In such cases, APs resorted to irony, joking, or oblique references to feelings of ambivalence, resignation or dissatisfaction with caring responsibilities that in some cases had shaped their lives over long periods:

Agnes, patient’s wife: It’s like there’s a tap running because I’ve seen it all before . . . I mean, well he’s either going to come round or he’s not [laughs]. Believe me, when you’ve put up with this for 13 years nothing fazes you or me.

[Interview with Agnes and the patient. Case 1.]

In some instances, APs alluded to their own wishes and needs when talking to practitioners. However, on no occasion did this type of communication generate discussion about support or respite for carers:

Kenneth, patient’s partner, to the occupational
therapist (OT): Can you not keep her in for a week?

OT: Pardon?

Kenneth: Can you not keep her in for a week?

OT: Not in this hospital but it depends – (. . .) if your partner couldn’t manage at home following this assessment we’d look at what options there are –

Kenneth: That was supposed to be a joke!

OT: Oh was it? [Laughs] I took it very seriously. (. . .) Well some people do—you know want to stay in the hospital but it’s not always the best place for their care so –

Kenneth: But it’s going to be difficult for her to move about at home.

[Recorded observation of the interaction between the OT, Kenneth and patient. Case 9.]

Kenneth attempted to backtrack from his direct request that his partner be kept in hospital by suggesting he had been joking, although his subsequent comment referred to her deteriorated mobility. The OT practitioner appeared to capture the seriousness of his repeated request, but reverted to a focus on the patient’s interests without exploring Kenneth’s personal concerns.

#### ED practitioners’ persuasive conversational strategies: APs “talked into being” as carers

Health professionals in our study had to assess cases without access to complete patient records, through pulling together rapidly-gleaned understandings of the patients’ living arrangements and support networks. When we explored the effects of time and resource pressures on ED decision-making in the wider study, our initial interpretation was that health professionals supposed that APs acted willingly as carers and that they were available to support patients’ discharge if this should be judged clinically viable. However, focused analysis of our interactional data indicated that ED practitioners did conversational work, when needed, to persuade ambivalent or resistant APs in that direction (Billig et al., 1988) and “talk them into being” (Heritage, 1984) as carers.

In some cases, health professionals’ efforts to facilitate discharge were concordant with the wishes of the patient and an AP. However, when there was overt or hinted-at discrepancy between an AP favoring admission (and reduced home care responsibilities) and an ED practitioner favoring discharge (with greater reliance on informal home care arrangements), the decision-making balance was invariably tipped by the weight of institutional authority represented by the practitioner. “Talking carers into being” took the form of health professionals listening to APs’ arguments for admission (rather than assuming they would support discharge), and immediately elaborating responses to counter these arguments. When APs introduced their own arguments such as fears about risks for the patient, ED practitioners responded by dismissing the validity of one concern after another, proposing solutions that focused on supporting patient mobility and safety.

This type of negotiation could be complicated by shifting AP–patient coalitions (Roscow, 1981), as shown in the example that follows. A 70-year-old patient, attending the ED after a dizzy spell and collapse, was accompanied at different times by two daughters and two sons (Case 3). There was divergence among the siblings regarding the advisability of their mother’s admission. Doris (Daughter 1) stated: “there’s no point keeping her in,” a view echoed by the patient. Elizabeth (Daughter 2), who lived closest to her mother, expressed concerns about risks of her falling at home. Elizabeth’s arguments were overcome by a consultant for care of the elderly who eventually secured the family’s collaboration with discharge.

In another case, an 86-year-old female patient affected by dementia resisted the idea of admission after a fall (Case 9, see Table 1). She and Kenneth, her 90-year old partner, were sometimes referred to by health professionals as a married couple, although each alluded to their “living in sin” relationship and basic independence. Kenneth, who had recently undergone a hernia operation, argued against the patient’s discharge home. Two ED practitioners enquired about their domestic arrangements with practical intent, but neither of them explored Kenneth’s willingness or ability to provide increased home care:

Junior doctor (to patient): Okay lovely and you’ve got your partner at home with you. Do you have any carers?

Patient: We have a lady who comes in and does a bit of vacuuming.

Junior doctor: Okay and you manage perfectly well between the two of you? Lovely. (Case 9. (Recorded observation of the interaction between the junior doctor, patient and Kenneth.)

Occupational therapist (OT to Kenneth): Have you got any questions or anything at the moment?

Kenneth: Well yes I’m rather concerned about her mobility –

OT: Her mobility, okay. What type of accommodation are you in? Is it a bungalow?

Kenneth: It’s a bungalow.

OT: A bungalow. Well that’s good news. (. . .) I’ve got lots of mobility aids and things like frames and I think probably – [to patient] you probably might need a frame to walk with just while your fracture is healing, just because it can give you some extra support.

(Case 9. Recorded observation of the interaction between the OT, Kenneth and patient.)

The junior doctor made a move to talk informal home care into being—“Okay and you manage perfectly well between the two of you? Lovely”—but the patient was eventually admitted because of her fracture. The above cases illustrate a parrying movement between the APs’ arguments about risk and health professionals’ assertions about safety. Issues underlying some APs’ objections to discharge—their own autonomy, living situation, health, and type of relationship with the patient—were invariably left unexplored.

## Discussion

Many individuals who accompany patients in health service visits do not regard themselves as carers (Hughes et al., 2013; Molyneaux et al., 2011). Some consider the carer label to be inappropriate for them, and others demonstrate difficulties coping in this role (Wingham et al., 2016). APs’ attempts to contribute to negotiations about caring may be “squeezed out” of decision-making by busy clinical schedules, lack of patient consent (Al-Janabi et al., 2016), and health professionals’ focus on the patient’s voice. A clinical criterion that discharge is in the patient’s best interest may challenge an AP’s view to the contrary. Tensions can emerge among the positions of all the parties involved, for example, when “social admissions” are considered due to gaps in home care or community services (Pinkney et al., 2016).

APs in our study gave clues to health professionals and researchers about situations that influenced their willingness or ability to comply with normative expectations about caring roles. APs’ shifting modes of engagement resonated with evidence from a study on patients with multiple sclerosis indicating that relatives and friends alternately “embraced, enforced, absorbed or rejected” identities as carers (Hughes et al., 2013). Interspersed with discussions about patient safety, tasks to be performed, and domestic adjustments, we also noted pivotal moments in which APs reflected on their relationships and prepared themselves for new roles.

Physician communication can have a critical impact when a person is transitioning from an AP to a carer (Karnieli-Miller et al., 2012). ED practitioners may use persuasive conversational strategies (Boyd & Heritage, 2006; Roy-Chowdhury, 2006), following an orderly sequence and avoiding overt conflict (Sharrock, 1979), to talk APs into taking on extended caring functions for system expediency. The drive for admission avoidance can school health professionals out of exploring the particularities of patients’ relationships with people accompanying them. APs, as well as patients and professionals, require time—a scarce commodity in EDs, globally (Chandler et al., 2015)—to communicate effectively and negotiate transitions in caring arrangements.

Health professionals and patients sometimes “cast” people as carers (Grimmer et al., 2004), although APs in our study rarely referred to themselves in this way. However, ED practitioners did not need to use the carer label to enlist APs’ support for discharge. It was sufficient, for purposes of supporting the resource-strained emergency system, to talk them into willingness to take on new or additional caring tasks. Such conversational persuasion brought pressure to bear on APs, sometimes triggering a sense of shame or guilt. Negative feelings about the self can provoke attempts to escape the shame-inducing situation, and the “hidden cost” of defensiveness and interpersonal separation (Tangney et al., 2007) may be detrimental to caring relationships.

Carers are not systematically addressed as co-clients and co-producers of the emergency system, and there is a need to detect “unacknowledged stress” and enable them to access available support (Al-Janabi et al., 2016; Georgiadis & Corrigan, 2017; Tangney et al., 2007; Wingham et al., 2016). “Carer-proofing” of decisions has been proposed to reduce strain on family carers for patients with long-term conditions (Al-Janabi et al., 2016). The concept of a “therapeutic alliance” has been used to explore relational factors including “intuitive supportive elements of the clinician–carer interaction” (Huff et al., 2015).

Shared decision-making has been defined as

an approach where clinicians and patients share the best available evidence when faced with the task of making decisions, and where patients are supported to consider options, to achieve informed preferences. (Elwyn et al., 2010)

It is striking that this definition does not take the APs’ presence and influence into account. A realist synthesis of evidence on integrated care for older people with complex needs called for further research on the involvement of relatives in shared decision-making (Bunn et al., 2018). A review of the studies on triadic medical consultations identified a need for physicians to establish role preferences of patients and companions (Laidsaar-Powell et al., 2013). Such consultations require careful balancing of focus on the patient and inclusion of the companion or family member, and for this, enhanced training for professionals in communication is critical (Cheung & Hocking, 2004; Karnieli-Miller et al., 2012; Manias, 2013). The persuasive conversational strategies (Boyd & Heritage, 2006; Roy-Chowdhury, 2006) observed in our study bear witness to the challenges, and also the potential for considering APs’ views within a shared decision-making framework (Elwyn et al., 2012). In the time- and resource-pressured ED environment, enhanced awareness of these issues may enable practitioners to develop more accurate appraisal of factors that can affect the patients’ forward care (Magdelijns et al., 2016).

The importance of a relationship-centered approach which includes members of the patient’s social network in decision-making has been emphasized in the literature (Adams & Gardiner, 2005; Nolan et al., 2003; Schneider et al., 2010). In areas of health care such as critical illness, family-centered care (FCC) is a central ethos, with associations reported between FCC and reduced family member anxiety (Hamzah & Sukarni, 2017). An integrative review of interventions intending to enable family involvement in the care of adults in acute hospital wards showed favorable impact on patient outcomes in seven studies (Mackie et al., 2018).

## Strengths and Limitations

Our focus on APs in ED settings fills a critical gap noted in the literature (Brown et al., 1998; Fry et al., 2015; Georgiadis & Corrigan, 2017) by providing in-depth, context-sensitive analysis of observational and interview data from ethnographic case studies. Data were collected from four acute hospitals using different emergency care models, with 56 interview participants, and analysis was informed by regular dialog with methodological and clinical experts.

The ethnic homogeneity of our sample is characteristic of the south-west England demographic. Further research is needed on socio-cultural and geographical variations in the profiles, relationships and expectations of patients and APs attending hospital emergency services. We did not collect data regarding the APs’ educational level or health literacy.

## Conclusion

Although contested definitions of carers and their roles have been noted in former research, effective recognition of, and response to, this mutability is still lacking in policy and practice. APs in our study reported many different types of relationship with patients and did not always conceive of themselves as carers. APs’ conversations with health professionals were influenced by system pressures in EDs as temporary stopping-places with a nationally-established time limit for decision-making. ED practitioners deployed conversational strategies to enlist APs in caring functions to support discharge.

The APs’ sense of shame or guilt could inhibit them from arguing openly about their limits to caregiving. Even in cases where APs were—seemingly, reluctantly—‘talked into’ extending their caring roles, health professionals did not follow up APs’ expressed concerns or mention support or respite available for carers.

A system-driven practice of talking APs into caring may generate pressure on people who are publicly undergoing shifts in their relationships with patients. There is a risk of negative outcomes for all involved if discharge arrangements fail or re-attendance ensues. In resource-stretched emergency services, interventions to avoid strain on people supporting patients can commence in the ED. By enquiring in an open way about APs’ own situations, experiences, and needs, health professionals can avoid the routinized attribution of patient–carer relationships that may prove socially shaming for APs to contest. Taking a relationship-centered or family-centered approach to caring could aid shared decision-making and attention to APs as clients of health systems in their own right.
